# Literary Notes

**Published:** 1910-12

**Authors:** 


### LITERARY NOTES

---

W. B. Saunders Company now have going through their presses a three-volume work on Practical Treatment, written by international authorities and edited by those able clinicians, Dr .John H. Musser and Dr. A. O. J. Kelly, both of the University of Pennsylvania.

In looking over the list of contributors we can come to but one conclusion—namely, that this work will undoubtedly take rank among the very best works on treatment extant. The names of the authors carry with them the positive assurance of thoroughness. Indeed, each chapter is a complete monograph, presenting the most recent therapeutic measures in a really practical way. As the general practitioner is required to know certain therapeutic measures more or less of a surgical nature, leading surgeons have been selected to present such subjects. This is an important feature and, to our knowledge, not included in any similar work.

In every case the men have been most aptly chosen for their respective tasks, and under the wise editorship of Drs. Musser and Kelly a work is being produced on treatment that will remain for many years a source of practical information, easily obtained and readily applied. The work will sell for $6.00 per volume, in sets only.

---

Physiologic Therapeutics, the new journal published by Dr. Henry R. Harrower, of Chicago, will celebrate the New Year with a special double number. We learn that several thousand extra copies will be printed and sent with the season's greeting to such physicians as may be interested in seeing this exponent of the progress in the non-medicinal methods of treatment. From the advance program which we have received it would seem that this number will be an especially fine one. Those of our readers who desire a copy should send a postal request to Dr. H. R. Harrower, Park Ridge, Illinois.

